# Risk of Obstructive Sleep Apnea and Echocardiographic Parameters

**DOI:** 10.5935/abc.20190181

**Published:** 2019-12

**Authors:** Adson Renato Leite, Diana Maria Martinez, Maria Luiza Garcia-Rosa, Erica de Abreu Macedo, Antonio José Lagoeiro, Wolney de Andrade Martins, Delvo Vasques-Netto, Cárita Cunha dos Santos

**Affiliations:** Universidade Federal Fluminense - Medicina Clinica, Niterói, RJ - Brazil

**Keywords:** Cardiovascular Diseases, Sleep Apnea, Obstruction, Indicators, Morbimortality, Heart Failure, Ecocardiography/methods, Polysonography/methods

## Abstract

**Background:**

Obstructive sleep apnea (OSA) is a chronic progressive disorder with high mortality and morbidity rate, associated with cardiovascular diseases (CVD), especially heart failure (HF). The pathophysiological changes related to OSA can directly affect the diastolic function of the left ventricle.

**Objectives:**

To assess the association of the risk of OSA, evaluated by the Berlin Questionnaire (BQ), and echocardiographic (ECHO) parameters related to diastolic dysfunction in individuals without HF assisted in primary care.

**Methods:**

A cross-sectional study that included 354 individuals (51% women) aged 45 years or older. All individuals selected were submitted to an evaluation that included the following procedures: consultation, filling out the BQ, clinical examination, laboratory examination and transthoracic Doppler echocardiography (TDE). Continuous data are presented as medians and interquartile intervals, and categoric variables in absolute and relative frequencies. The variables associated with risk of OSA and at the 0.05 level integrated the gamma regression models with a log link function. A value of p < 0.05 was considered an indicator of statistical significance. Exclusion criteria were presence of HF, to fill out the BQ and patients with hypertension and obesity not classified as high risk for OSA by other criteria. All individuals were evaluated on a single day with the following procedures: medical appointment, BQ, laboratory tests and ECHO.

**Results:**

Of the 354 individuals assessed, 63% were classified as having high risk for OSA. The patients with high risk for OSA present significantly abnormal diastolic function parameters. High risk for OSA confirmed positive and statistically significant association, after adjustments, with indicators of diastolic function, such as indexed left atrium volume LAV-i (p = 0.02); E’/A’ (p < 0.01), A (p = 0.02), E/A (p < 0.01).

**Conclusion:**

Our data show that patients at high risk for OSA present worsened diastolic function parameters measured by TDE.

## Introduction

Obstructive sleep apnea (OSA) is a chronic progressive disorder with high mortality and morbidity rate and is associated with cardiovascular diseases (CVD), including heart failure (HF).^1^ The physiopathological interaction between OSA and cardiovascular disease is complex and involves sympathetic activation, oxidative stress and inflammation, endothelial disfunction and disfunction of the Circadian clock gene.^2-4^

Besides polysomnography, considered the gold standard for the diagnosis of OSA, there are different scales which do not diagnose the disease, but indicate the people at risk, among which is the Berlin Questionnaire (BQ).^5^ A meta-analysis published in 2017 estimated that the sensitivity of the Questionnaire to detect OSA was 76%, 77% and 84% and its specificity was 59%, 44% and 38% for patients with mild, moderate and severe OSA, respectively. It is necessary to point out the adequate sensitivity which enables the BQ as a tracking too, making early diagnosis of OSA possible.^6^

The prevalence of diastolic disfunction in patients with OSA ranges from 23% to 56% and there is a dose-response relation between the severity of diastolic disfunction and the severity of strong physiopathological basis demonstrated for a continuum of diastolic disfunction and heart failure in their two phenotypes, which means a greater risk for these patients to develop HF. The association of OSA with diastolic disfunction was observed even in its initial stages.^7^

We have not yet found studies of the association of characteristic echocardiographic parameters of diastolic disfunction and the presence of the risk of OSA in patients with no signs or symptoms of heart failure.

The purpose of this study was to assess the association of the risk of OSA and echocardiographic parameters related to diastolic disfunction in patients without HF assisted by “*Programa Médico de Família*” (PMF - Family Doctor Program) in the city of Niterói.

## Methods

A cross-sectional study that integrates the DIGITALIS STUDY and included 633 individuals (51% females) between 45 to 99 years of age enrolled in the PMF, Niterói, RJ. The data were obtained from July 2011 to December 2012. The methodology applied was previously described.^8^

All individuals selected for the study were evaluated on a single day. The evaluation included the following procedures: filling out the questionnaire, consultation and clinical examination, laboratory tests and transthoracic Doppler echocardiography (TDE).

**Figure 1:**
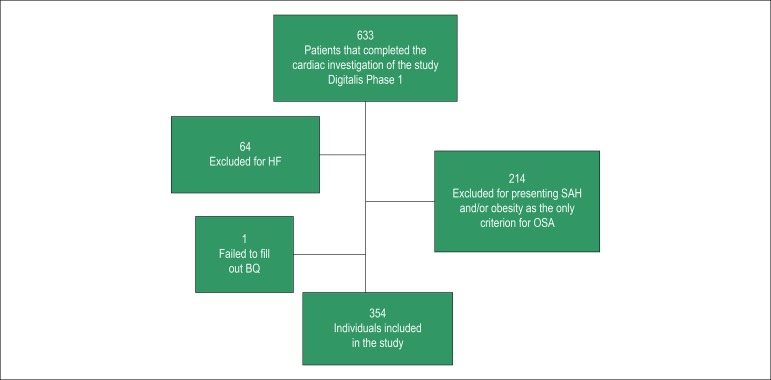
Flowchart of sample selection. BQ: Berlin Questionnaire; HF: heart failure; HBP: high blood pressure or arterial hypertension; OSA.

Of the 633 participants examined by the DIGITALIS STUDY, 64 were excluded for having been diagnosed with HF or for not having answered the BQ completely, and 214 for having hypertension or being obese and not having been classified as having risk of OSA by other criteria. For the present analysis, 354 individuals were included (figure 1).

The TDE scans were performed by two echocardiographers who had no previous knowledge of the results of the other tests, using two pieces of equipment (Cypress 20 Acuson/Siemens EUA/AU-3 Partner, Esaote - Italy). The tests were performed according to the recommendations for quantification of chambers of the American Society of Echocardiography (ASE) and the European Association of Echocardiography (EAE). Systolic function was evaluated by measuring the left ventricular ejection fraction (LVEF) by the Simpson’s method.^9^

The participants were categorized into positive or negative for the risk of OSA based on their answers to the individual items of the QB and their total score in the categories of symptoms.^5^

### Statistical analysis

This was performed with the SPSS v 21.0 (Chicago, Illinois, USA). Continuous data are presented as medians and interquartile ranges and categorical variables as relative and absolute frequencies. The differences between groups with and without risk of OSA were tested by the non-parametric Mann-Whitney test and the categorical variables by the qui-square test with correction of continuity and Fisher’s exact test, when necessary. The variables that presented an association with the risk of OSA at a 0.05 level integrated the gamma regression models with a log link function. As echocardiographic parameters are very correlated among each other, it was chosen to adjust a model for each parameter (outcome) associated with the presence or absence of risk of OSA in the preliminary analysis at a level of 0.05. Exposed and non-exposed coefficient exponentials are interpreted as the result of the arithmetic means of the outcome. A value of p < 0.05 was considered an indicator of statistical significance.

### Ethical considerations

This study was conducted according to the principles established in the Declaration of Helsinki, revised in 2000 (Scotland, 2000). The protocol for the study was approved by the Institution’s Research Ethics Committee under code number CAAE:0077.0.258.000-10.

## Results

Of the 354 individuals analyzed, 63% were classified as having a risk of OSA. Table 1 presents the clinical characteristics according to the presence of the risk of OSA. Individuals with risk were mostly women, older, with higher BMI, glucose, uric acid and triglyceride levels, urine albumin-to-creatinine ratio and blood pressure. The patients with risk of OSA, compared to the ones with no risk, presented greater abnormalities of the diastolic function parameters: LAV-i (+), DT (+), E(-), E’/A’ (+), E/E’ (-), A(+), E/A (+), PPEI (+) and VIS (+), which may indicate a less effective diastolic function. Such differences were statistically significant (Table 2).

**Table 1:** Median with interquartile* range or absolute and relative frequency ** of clinical characteristics according to the presence of high risk for OSA modified***

	High risk of OSA modified*		Value
	Yes n = 223	No n = 131	
**Gender**			**0.01**
Male	79 (35.4)	66 (50.4)	
Female	114 (64.6)	65 (49.6)	
Age (in years)	57.0 (51.0-63)	54.0 (49.0-61.0)	0.01
BMI kg/m^2^	29.4 (26.1-33.0)	24.6 (22.4-27.3)	< 0.01
Glucose (mg/dL)	102.5 (92.0-117.2)	97.0 (88.0-108.0)	< 0.02
Urea (mg/dL)	31.0 (26.0-37.0)	31.0 (25.2-36.0)	0.69
Creatinine (mg/dL)	0.82 (0.71-0.99)	0.85 (0.74-0.96)	0.56
Uric acid (mg/dL)	5.6 (4.4-6.6)	4.7 (3.9-5.6)	< 0.01
Cholesterol (mg/dL)	219.0 (193.0-250.0)	213.0 (187.0-239.0)	0.17
LDL-cholesterol(mg/dL)	135.8 (117.7-163.5)	134.1 (107.3-159.1)	0.19
HDL-cholesterol (mg/dL)	41.0 (51.5 (63.0)	55.0 (44.0-63.0)	0.23
Triglycerides (mg/dL)	126.5 (96.0 (183.7)	106.0 (73.0-153.0)	< 0.01
Urine albumin-to-creatinine ratio	9.9 (5.7-22.3)	7.7 (4.7-13.6)	< 0.01
Mean heart rate (bpm)	71.0 (63.0-80.0)	69.0 (62.5-76.5)	0.19
Systolic arterial pressure l (mmHg)	137.33 (122.5-152.0)	122.0 (113.3-129.5)	< 0.01
Diastolic arterial pressure (mmHg)	84.0 (76.3-92.67)	75.5 (70.3-80.7)	< 0.01
**Myocardial infarction**			
Yes	9 (4.0)	4 (3.1)	0.86
No	214 (96.4)	127 (96.9)	
**Stroke**			
Yes	11 (4.9)	1 (0.8)	0.07
No	212 (95.1)	130 (99.2)	

OSA: obstructive sleep apnea; BMI: body mass index; bpm: beats per minute.

* Differences tested by the Mann-Whitney test;

** Differences tested by Pearson's qui-square test with continuity correction or Fisher's exact test when necessary;

*** Individuals who were classified as at risk only in category 3 were excluded (Adapted from Netzer et al., 1999).^5^

**Table 2:** Median with interquartile* range or absolute and relative frequency ** of echocardiographic parameters according to the presence of high risk of OSA modified

	High risk of OSA modified		p value
	Yes	No	
ILAD (cm/m^2^)	1.9 (1.7-2.1)	1.9 (1.7-2.0)	0.37
ILAV-i (ml/m^2^)	21.1 (17.7-24.9)	19.9 (16.8-22.7)	0.01
DT (ms)	228.0 (186.0-261.0)	200.0 (174.0-228.0)	< 0.01
E' (cm/s)	10.0 (8.0-12.0)	11.5 (9.0-13.0)	< 0.01
E'/A'	0.83 (0.64-1.20	1.14 (0.80-1.37)	< 0.01
E/E'	6.4 (5.4-7.8)	6.0 (5.0-7.0)	0.02
E (cm/s)	63.0 (53.0-76.0)	66.1 (54.0-75.0)	0.31
A (cm/s)	68.0 (56.0-81.9)	58.0 (48.0-68.0)	< 0.01
E/A	0.93 (0.7-1.2)	1.18 (0.9-1.4)	< 0.01
ILVM (g/m^2^)	89.4 (77.3-103.6)	88.7 (74.4-102.0)	0.49
IFDV (ml/m^2^)	62.08 (53.5-68.7)	63.8 (54.0-72.3)	0.15
RWT (mm)	0.3 (0.3-0.4)	0.3 (0.3-0.4)	0.28
IPWT (mm)	8.0 (7.0-9.0)	8.0 (7.0-8.0)	0.03
ILVDD (mm)	49.0 (46.0-51.0)	48.0 (45.0-51.0)	0.53
IVS (mm)	8.0 (7.0-9.0)	8.0 (7.0-9.0)	0.02

ILAD: indexed left atrial diameter; LAV-i: indexed left atrial volume; WT:E' wave deceleration time; E: early diastolic mitral annular velocity; E'/A': early diastolic mitral flow velocity; A:atrial contraction; ILVM: indexed left ventricular mass; IFDV: indexed final diastolic volume; RWT: relative wall thickness; IPWT: indexed posterior wall thickness; ILVDD: indexed diastolic left ventricular diameter; IVS: intraventricular septum.

* Differences tested by the Mann-Whitney test;

** Differences tested by Pearson's qui-square test with correction by Fisher's exact test when necessary.

****** Individuals who were classified as at risk only in category 3 were excluded (Adapted from Netzer et al., 1999).^5^

The exponentials of the coefficients for each gamma regression model are presented in table 3. In all cases, the exponentials of the coefficients were adjusted for gender, age, BMI, fasting glucose, triglycerides, uric acid, urine albumin-to-creatinine ratio and systolic and diastolic blood pressure in their continuous forms. Association of the high risk of OSA with less effective diastolic function was confirmed for: LAV-i (+), E/A (+), E’/A’(+), A (+) association with DT (+) E’ (+), which reached a significance of 0.10 (Table 3).

**Table 3:** Exponentials of gamma regression** adjusted coefficients* of the presence of high risk of OSA (yes/no)***

	Exponential of the adjusted coefficient	p value
LAV-i (ml/m^2^)	1.10 (1.02-1.18)	0.02
TD (ms)	1.05 (0.99-1.11)	0.10
E'	1.05 (0.99-1.11)	0.10
E'/A'	0.87 (0.72-0.96)	< 0.01
E/E'	1.01 (0.94-1.09)	0.81
A	1.10 (1.02-1.18)	0.02
E/A	0.86 (0.79-0.94)	< 0.01
IEPP (mm)	1.02 (0.98-1.06)	0.24
SIV (mm)	1.02 (0.98-1.06)	0.42

LAV-i: indexed left atrial volume; WT: E' wave deceleration time; E: early diastolic mitral annular velocity; E'/A': early diastolic mitral flow velocity; A: atrial contraction; IPWT: indexed posterior wall thickness; IVS: intraventricular septum.

* For each regression model whose outcome was an echocardiographic parameter, exponentials of the coefficients were adjusted for gender, age, BMI, fasting glucose, triglycerides, serum uric acid, urine albumin/creatinine ratio and systolic and diastolic arterial pressure in their continuous forms (mmHg),

** Gamma regression with log link function;

*** Berlin Questionnaire.

## Discussion

The present study evaluated the presence of abnormalities on TDE, associated with diastolic disfunction, in individuals without signs or symptoms of HF, according to the presence of risk of OSA. The BQ was used as a tool and the individuals with obesity and high blood pressure who did not present other criteria for OSA were excluded. In primary care, selective methods for OSA are more easily applied than standard polysomnography, being useful in the stratification of risk, as they have lower costs and are easily accessible.^7^ Using the BQ in the population assisted in primary care programs, such as the “*Médico de Família*” program, would help to select patients at risk for OSA, who should then be referred for TDE and polysomnography investigation.

OSA is related to different physiopathological mechanisms triggered by hypoxia and sleep fragmentation, involving sympathetic hyperactivity, inflammation, endothelial disfunction and oxidative stress, among other factors leading to arterial hypertension, atrial fibrillation, stroke and HF outcomes.^10^

Various studies have demonstrated alterations of different markers of diastolic function of the LV in patients with OSA as an indexed increase in left atrial size (LAV-i),^11,12,13^ altered E/A ratio,^14,15^ early diastolic mitral annular velocity (E’)^16,17^ and increase in E/E ratio.^14,18^ Our data show alterations in some of these markers: LAV-i, E’/A’ ratio, A wave, E’ and E/A ratio in patients at risk for OSA.

We observed that LAV-i, a marker of diastolic disfunction, presents a strong association with the presence of high risk of OSA, identified by the BQ, regardless of the presence of hypertension or obesity, when not associated with an indicator of the risk of OSA. Wachter et al.^3^ investigated if OSA affects diastolic function in a primary care cohort and observed that diastolic function is independently associated with OSA in patients with cardiovascular risk factors.^3^ Gottlieb et al. observed that in patients without HF and coronary arterial disease, the presence of OSA was an independent HF predictor in men and not in women.^19^ In another study, Usui et al.^20^ demonstrated that the severity of OSA may contribute directly to LV diastolic disfunction regardless of LV geometry, arterial stiffness, obesity and is associated with cardiovascular risk factors.^20^

In patients with controlled arterial hypertension, Lisi et al.^21^ observed that mild to moderate OSA, diagnosed by polysomnography, is associated with diastolic disfunction, regardless of age, gender and mean arterial blood pressure levels and in the absence of concentric left ventricular hypertrophy or increased left atrium. The authors suggest that nocturnal hypoxemia could be the key factor for the development of diastolic disfunction.^21^

Hypertension is the main cause of diastolic disfunction and is also one of the biggest consequences of OSA.^22^ Two studies excluded obese individuals from the analysis^20,23^ and at least one excluded obese and hypertensive individuals.^23^ The two articles studied solely individuals with OSA and compared the moderate OSA group with the one with severe OSA. In both studies, the E/A association was statistically significant. In the study of Imai et al.,^23^ LAV-i and E/E’ ratio were significantly bigger in the severe OSA group. The data from these two studies show that the association of the OSA with abnormal diastolic function may occur in non-obese and non-hypertensive individuals. Due to the high prevalence of these two conditions, in the present study, it was not possible to exclude them from the analysis to confirm the independent association and the risk of OSA and the indicators of diastolic disfunction.

The present study evaluated the contribution of several echocardiographic parameters, which represent, with bigger reliability, the structural or cardiac function abnormalities that may be associated with the diagnosis of OSA. LAV-i. TD, E/A ratio, E’/A’ ratio and A wave abnormalities in individuals with OSA indicated a less effective diastolic function in patients with sleep disorders, compatible to findings that defined OSA through polysomnography.

### Limitations

The BQ does not confirm the OSA and only points out those patients at risk for the syndrome, with reduced sensitivity and specificity, questionable reproductivity, because the perception and documentation of what is informed may not be precisely estimated, since it involves limitations resulting from the level of literacy or pre-existing cerebral vascular conditions of the informant, making it difficult to understand the BQ, especially by the elderly. Due to the limitation of resources and because it is a tracking study, each patient was examined by only one echocardiographer, preventing inter or intra-observer concordance examination. Despite these limitations, the results according to the TDE parameters among the risk groups were in line with those of the literature.

Because it is a cross-sectional study, it was not possible to establish a causal link. Despite having excluded from the analysis hypertensive and obese individuals that who did not meet any other criterion for the risk of OSA according to the BQ, those at risk presented higher mean BMI, systolic and diastolic arterial pressure, which notwithstanding the control (inclusion in multiple models) still may have caused residual confounding.

## Conclusions

Evaluation of the association of OSA and the presence of structural and functional cardiac abnormalities obtained by the TDE can contribute to a discussion about the adoption of the BQ in the community, to select individuals with cardiovascular risk that should undergo TDE, despite its limitations.

This strategy of fast execution may be easily incorporated into the routine of assessment of patients with risk factors for the development of HF, but it still needs a detailed analysis and long-term follow-up for its definitive prescription.
